# Permafrost thaw subsidence, sea-level rise, and erosion are transforming Alaska’s Arctic coastal zone

**DOI:** 10.1073/pnas.2409411121

**Published:** 2024-12-03

**Authors:** Roger Creel, Julia Guimond, Benjamin M. Jones, David M. Nielsen, Emily Bristol, Craig E. Tweedie, Pier Paul Overduin

**Affiliations:** ^a^Department of Physical Oceanography, Woods Hole Oceanographic Institution, Falmouth, MA 02543; ^b^Department of Applied Ocean Physics and Engineering, Woods Hole Oceanographic Institution, Falmouth, MA 02543; ^c^Institute of Northern Engineering, University of Alaska, Fairbanks, AK 99775; ^d^Department of Climate Variability, Max Planck Institute for Meteorology, Hamburg 20146, Germany; ^e^Center for Earth System Research and Sustainability, University of Hamburg, Hamburg 20146, Germany; ^f^Marine Science Institute, College of Natural Sciences, University of Texas at Austin, Austin, TX 78373; ^g^Department of Biological Sciences, University of Texas at El Paso, El Paso, TX 79902; ^h^Department of Environmental Science and Engineering Program, University of Texas at El Paso, El Paso, TX 79902; ^i^Alfred Wegener Institute Helmholtz-Centre for Polar and Marine Research, Permafrost Section, Potsdam 14401, Germany

**Keywords:** permafrost thaw subsidence, sea-level rise, coastal erosion, Arctic, climate hazards

## Abstract

Arctic shorelines are vulnerable to climate change impacts as sea level rises, permafrost thaws, storms intensify, and sea ice thins. Seventy-five years of aerial and satellite observations have established coastal erosion as an increasing Arctic hazard. However, other hazards at play—for instance, the cumulative impact that sea-level rise and permafrost thaw subsidence will have on permafrost shorelines—have received less attention, preventing assessments of these processes’ impacts compared to and combined with coastal erosion. Alaska’s Arctic Coastal Plain (ACP) is ideal for such assessments because of the high-density observations of topography, coastal retreat rates, and permafrost characteristics, and importance to Indigenous communities and oilfield infrastructure. Here, we produce 21st-century projections of Arctic shoreline position that include erosion, permafrost subsidence, and sea-level rise. Focusing on the ACP, we merge 5 m topography, satellite-derived coastal lake depth estimates, and empirical assessments of land subsidence due to permafrost thaw with projections of coastal erosion and sea-level rise for medium and high emissions scenarios from the Intergovernmental Panel on Climate Change’s AR6 Report. We find that by 2100, erosion and inundation will together transform the ACP, leading to 6-8x more land loss than coastal erosion alone and disturbing 8-11x more organic carbon. Without mitigating measures, by 2100, coastal change could damage 40 to 65% of infrastructure in present-day ACP coastal villages and 10 to 20% of oilfield infrastructure. Our findings highlight the risks that compounding climate hazards pose to coastal communities and underscore the need for adaptive planning for Arctic coastlines in the 21st century.

## Significance

Arctic coastlines are changing rapidly due to the combination of permafrost thaw subsidence, sea-level rise, and erosion. These processes have received unequal attention, and their compound impact remains poorly understood. Alaska’s Arctic Coastal Plain (ACP) is ideal for addressing this knowledge gap due to the region’s relatively abundant observational data and importance to Indigenous communities, socioeconomics, and geopolitics. We present projections of future ACP evolution that include subsidence, sea-level rise, and erosion. By 2100, unless coasts respond differently to future change, these compound effects may transform 6-8x more land than erosion alone may impact. Our findings underscore that coastal communities will need support to adapt to the paradigm shift that Arctic coastlines may soon undergo.

Climatic warming is causing rapid changes to Arctic coastal regions. In the last four decades, Arctic temperatures have increased at four times the global mean (1). Rising temperatures are accompanied by a cascade of Earth system consequences: Land ice is melting; sea ice extent is diminishing; open water periods are lengthening; sea level is rising; coastal erosion is intensifying; and frozen ground is thawing (2). Projections of climate evolution indicate that these trends will persist throughout the 21st century and that the severity of the resulting impacts to coastal communities (3)—and the organic carbon (OC) and contaminants that get mobilized—will depend on the speed at which anthropogenic atmospheric greenhouse gas accumulation is reduced (4). In Alaska, and the Arctic as a whole, present-day climate changes are amplifying long-standing threats and introducing additional challenges to community adaptation—particularly coastal Indigenous communities. This heightened threat is in part because the compounding nature of these changes produces nonlinear increases in coastal hazards (5).

Coastal erosion, subsidence from permafrost thaw (hereafter, permafrost subsidence), and sea-level rise have each individually received attention as important threats to Arctic landscapes. Thanks to repeat aerial surveys starting in mid-20th century (6), rates of Arctic coastal erosion are known to be among the highest in the world and to have accelerated throughout the last ∼80 y (7). Two forces drive this coastal permafrost erosion: mechanical thermo-abrasion from wave action and thermo-denudation from insolation and summer warmth (8, 9). Recent observations from a geographic spread of coastal monitoring sites provide a glimpse of how Arctic System changes are intensifying permafrost coastal dynamics. For instance, along the US Beaufort Sea coast, shoreline change increased 80% from the 1970s to 2000s and 133% from the 2000s to 2010s (2). Coastal erosion has significant impacts on infrastructure and property (10) as well as on natural resource-based land uses (11).

Permafrost subsidence has also been identified as a coastal threat. Permafrost-related vertical land motion occurs on a range of scales. Seasonal variations in active layer thickness can lead to decimeter-scale cycles of heave and subsidence (12). Fire and human-induced disturbances to tundra environments can also trigger local subsidence rates approaching a decimeter per year, and these rates can persist for decades (13). Human-related disturbances are often associated with infrastructure, which is one of the landscape types most impacted by Arctic climate evolution: Through thermokarst, active layer thickening, mass movement, and other warming-related hazards, permafrost degradation undermines roads, damages pipelines, and destabilizes building foundations (14).

Over broader regions, repeated measurement of permafrost elevations began around mid-20th century to identify centimeter-scale annual subsidence of ice-rich permafrost (18). This land motion has been attributed to late-season thawing of ground ice driven by warming near-surface air temperatures (19)—a pattern that has accelerated in the 21st century (20). Such “isotropic” permafrost thaw has been resolved spatially via interferometric synthetic aperture radar (InSAR) (12, 13), which, when paired with differential GNSS (global navigation satellite system) or other in situ observations, can precisely constrain interannual permafrost subsidence.

**Fig. 1.**
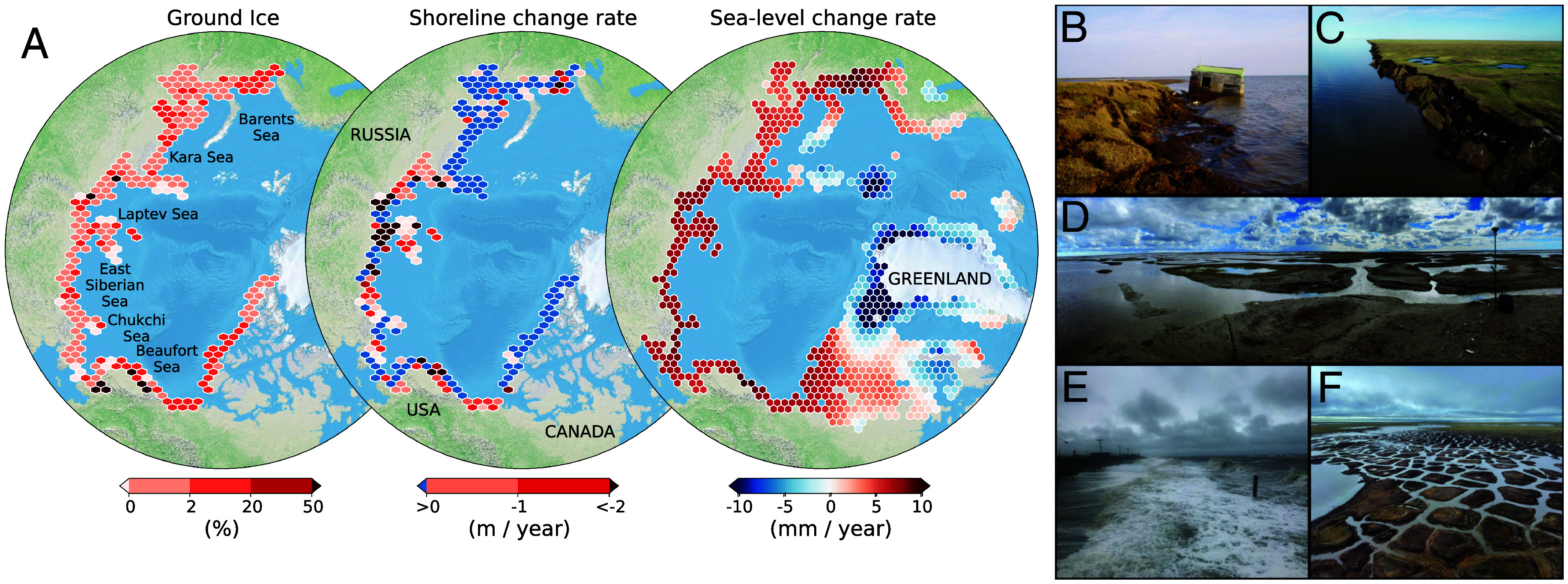
Variability of Arctic ground ice, shoreline change, and sea level. (*A*) Ground ice and shoreline data are from the ACD database (15). Sea-level change rates (2020 to 2100 mean) are for the IPCC-AR6’s mid-level emissions scenario (SSP2-4.5) (16, 17). (*B*) Erosion undercuts an Iñupiaq cabin, Elson Lagoon, Alaska. (*C*) Cabin-sized permafrost blocks collapse into the Beaufort Sea, Drew Point. (*D*) Seawater drowns ice-wedge polygonal tundra, Ikpikpuk Delta, Alaska. (*E*) Storm threatens infrastructure, Utqiagvik, Alaska. (*F*) Marine flooding degrades permafrost, Point Lonely, Alaska. Images from coauthor BMJ.

Sea-level rise regularly features in Arctic threat assessments as a process that will increase the risks posed by extreme events such as ocean surges (21). The projected impacts of sea-level rise on Arctic shorelines are spatially heterogeneous. Regions near areas of ice mass unloading—e.g., Arctic Canada, Greenland, Southeast Alaska, Western Siberia—will undergo net sea-level fall due to glacial isostatic adjustment (16, 17). Some of these areas are gaining ground as modern ice retreat uncovers new coastline (22); other areas gain ground as glacial erosion speeds deltaic progradation (23). Arctic communities far from rapid isostatic uplift, however, are routinely identified as being at high risk of sea-level rise-driven flooding (2, see Fig. 1). For instance, by 2100, sea level at Prudhoe Bay, Alaska, is projected under mid/high emissions scenarios to reach 0.97 m (median, 0.73 to 1.26 m, 90% credible interval)/1.19 m (0.88 to 1.56 m) above present levels (16, 17). For a few Alaskan communities, this flooding risk has been paired with estimates of permafrost thaw potential to generate inundation projections (24). Alaskan sea-level projections have also been paired with ground settlement indices and erosion projections to develop coastal hazard indices for the Alaskan North Slope (25). In the larger Arctic coastal hazard community, there is broad consensus that regions undergoing high rates of coastal erosion, permafrost subsidence, and sea-level rise are at greatest risk of climate impacts. However, coastal permafrost studies have not projected the compounding effect that these processes will have on Arctic shorelines and low-lying tundra landscapes (2, 7).

**Fig. 2.**
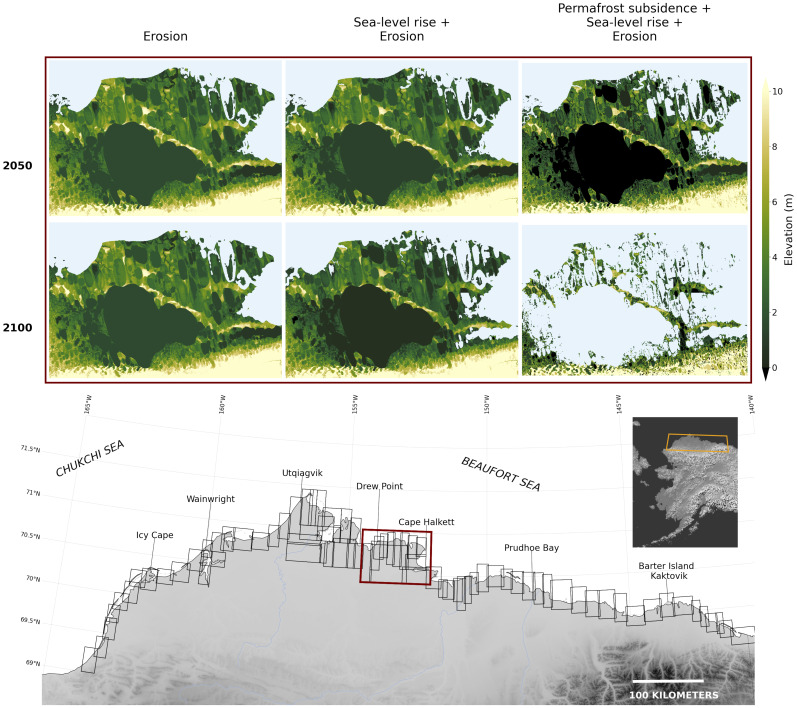
Evolution of the Teshekpuk Lake region in the 21st century. Black boxes in the *Bottom* panel denote Arctic Coastal Plain subregions in which analysis was performed. *Inset* maps indicate 2050 and 2100 time slices for erosion (*Left*), erosion plus sea-level rise (center), and erosion plus sea-level rise plus permafrost subsidence (*Right*). Colormap denotes topography; light blue is ocean. Projections from a mid-range emissions scenario (SSP2-4.5) are shown.

We address this knowledge gap by producing projections of 21st-century Arctic shoreline position that account for coastal erosion, permafrost subsidence, and sea-level rise. We focus on Alaska’s Arctic Coastal Plain (ACP, Fig. 2), which has an abundance of ice-rich permafrost and among the highest rates of sea-level rise in the Arctic (16, 17). The ACP is a low-elevation, low-relief landscape that rises southward from the Arctic Ocean to intersect the foothills of the Brooks Range at 120 to 200 m asl. Over 60,000 km^2^ in area, the terrain is composed of ice-bonded Quaternary marine, fluvial, and eolian sediments reworked extensively by thermokarst processes. Permafrost sediments at the coast and in the low-lying hinterland tend to be extremely ice-rich, approaching a volumetric ice content of 80% when ice wedges are included (26). Constraining ACP shoreline position is uniquely possible because of high data density, including high-resolution topographic maps, numerous observations of permafrost landscape characteristics, and a long history of coastal retreat estimation. We first join a 5 m ACP digital elevation model with InSAR-derived lake depth estimates (27). We then develop an algorithm to erode the ACP following the erosion projections of (28), which are based on scenarios defined by the Intergovernmental Panel on Climate Change’s AR6 Report. This algorithm includes periodic coastal smoothing to simulate observed coastal erosion dynamics and storm-driven sediment redistribution. Next, we produce projections of ACP permafrost subsidence by compiling interannual subsidence measurements from lowlying Arctic regions (*SI Appendix*, Fig. S1) and mapping them onto an ACP landform classification dataset (29).

We combine these subsidence and erosion estimates with relative sea-level projections from the Fifth National Climate Assessment (30) to project coastal evolution for the 21st century. With these simulations, we quantify land loss due to erosion, permafrost subsidence, and sea-level rise, assess the relative importance of each driver of coastal change over time, and project when land loss due to the combination of inundation and erosion will surpass land loss driven by erosion alone. We then estimate the fraction of present-day ACP infrastructure that the landscape change we project would damage without mitigation measures. Finally, we compute the amount of OC that the projected land loss could disturb, where disturb means mobilize through erosion or alter via downward diffusion of seawater into sediment.

## 1. Results and Discussion

### 1.1. Land Loss.

**Fig. 3.**
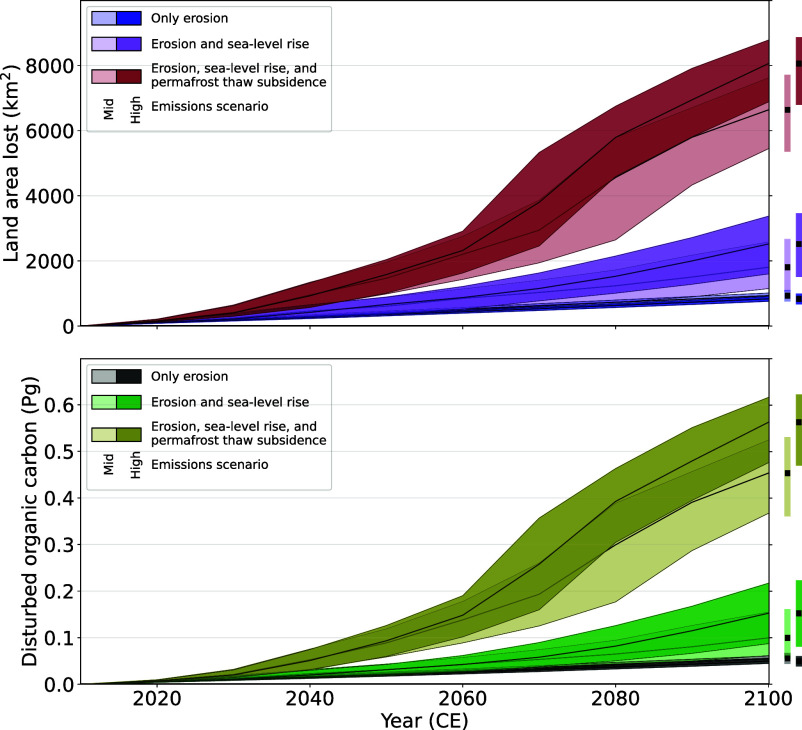
Projected 21st-century land loss and organic carbon disturbance on Alaska’s Arctic Coastal Plain (ACP). Light/dark blue (gray) lines and envelopes (17th to 83rd quantile) represent land loss (organic carbon disturbance) due to erosion under a medium/high emissions scenario (SSP2-4.5/SSP5-8.5); purple (green) lines, the combined effect of erosion and sea-level rise; Red (beige) lines, the combined effect of erosion, permafrost subsidence, and sea-level rise.

We find that under a medium emissions scenario, the ACP loses 1,469 km^2^ (989 to 1,956 km^2^, 68% credible interval) of land by 2050 and 6,638 km^2^ (5,446 to 7,620 km^2^) by 2100 (Fig. 3)—an area larger than Trinidad and Tobago. With high emissions, those projections increase to 1,581 km^2^ (1,014 to 2,036 km^2^) of land by 2,050 and 8,059 km^2^ (6,886 to 8,778 km^2^) of land by 2100—an area nearly the size of Puerto Rico.

We compare our projections to existing regional tallies of land loss from the combination of erosion and inundation. Merging sediment flux measurements at 48 sites with historical observations, (31) estimated Beaufort Sea land loss is 2.03 km^2^/y. Teshekpuk Lake Special Area, with ∼140 km of shoreline, lost 0.65 km^2^/y from 1979 to 2002, while a ∼40 km length of shoreline from Sagavanirktok River delta to Point Thomson lost 0.76 km^2^/y from 2006 to 2010 (32). These latter rates, scaled to the full ACP shoreline, would equal ∼9 and 38 km^2^/y land loss, respectively. Our land loss rates at 2020 fall within these existing rates of ACP land area loss, but under medium or high emissions scenarios will exceed existing rates by midcentury (Fig. 3).

Permafrost subsidence amplifies land loss. Accounting for permafrost subsidence and sea-level rise in addition to erosion leads to mean additional land loss of 4,832(5,539) km^2^ under medium(high) emissions (Fig. 3). The difference between projections that only include erosion versus those that include both erosion and inundation is stark: Including inundation increases land loss six-fold under medium emissions and eight-fold under high emissions. Including inundation also amplifies rates of land loss. With only erosion, mean 21st-century ACP land loss never exceeds 10.8 km^2^/y. With erosion and sea-level rise, mean land loss rises from 19(22) km^2^/y by 2050 to 33(54) km^2^/y by 2100 under medium(high) emissions. With erosion, sea-level rise, and permafrost subsidence, land loss accelerates to 64 km^2^/y by 2050 in either emissions scenario and peaks at 173(209) km^2^/y by 2072/2076 under medium(high) emissions.

ACP land loss accelerates in the 21st century because linear subsidence increases drive nonlinear inundation increases. The ACP is covered with lakes and drained lake basins, the beds of which are typically not more than a few meters above sea level. By midcentury, as permafrost subsidence lowers the landscape toward sea level, those lakes connect with the ocean and their margins begin to erode, exposing more lakes to inundation. This fractal behavior can in some settings stabilize shorelines by dampening erosion (33). However, the fractal shoreline behavior modeled here does not depend on erosion: the ∼6,000 km^2^ more land lost when permafrost subsidence is included occurs with no change in erosion rates, and the difference between medium versus high emissions scenario erosion rates has only a modest impact on that result. Rather, land loss accelerates because of the ACP’s >13,000 lakes and drained-lake basins, a low-lying, high-relief system that last flooded between 70 and 115 kyr ago, the last time Earth was substantially warmer than present (34).

### 1.2. Impacts to Society.

**Fig. 4.**
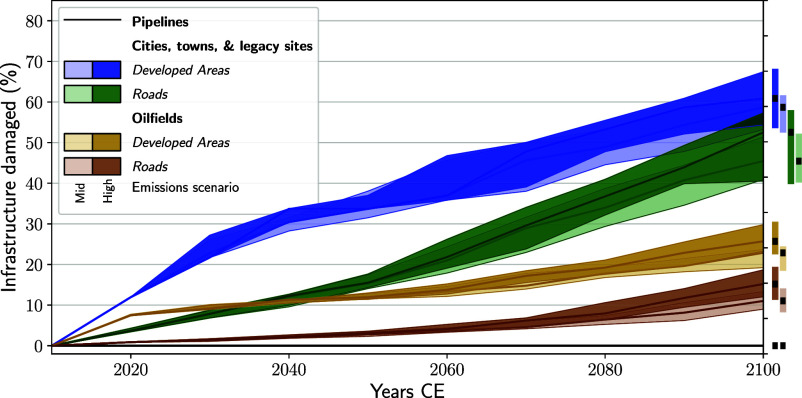
Present-day infrastructure damaged by coastal change on Alaska’s Arctic Coastal Plain (ACP) over the 21st century. Light/darker blue and green lines and envelopes represent developed areas of ACP cities, towns, and legacy sites and related roads damaged by coastal change under medium/high emissions scenarios (SSP2-4.5/SSP5-8.5); brown/yellow lines, the same but for oilfields. The black line denotes oil pipeline damages. Envelopes are 17th to 83rd quantile.

We quantify the fraction of present-day infrastructure that erosion and inundation would damage over the 21st century without mitigation measures. Under medium emissions, erosion and inundation by 2100 damage 59(53 to 61)% of developed areas and 45(41 to 51)% of roads in ACP cities, towns, and legacy sites, while in ACP oilfields, 23(19 to 24)% of developed areas, 11(9 to 13)% of roads, and 0% of pipelines are damaged. A high emissions scenario increases these projections modestly (Fig. 4). Some infrastructure damage happens before other damage. Developed areas are impacted at highest rates before 2040. Roads connecting cities, towns, and legacy sites are impacted most after 2050, while oilfield-related roads are minimally impacted. These differences reflect the elevational and geographic distributions of each infrastructure type: Developed areas tend to occupy low-lying coastal sites (e.g., Prudhoe Bay), while roads span a range of elevational terrains and pipelines stretch directly inland. The largest uncertainty in future infrastructure damages is human action. The damages we project could be amplified if more infrastructure is built in low-lying coastal areas, or lessened if industries and governmental agencies commit to protect or relocate the infrastructure currently under threat. We do not account for this uncertainty in action.

### 1.3. Organic Carbon Impacts.

We next quantify the OC that 21st-century ACP coastal change could disturb. Erosion-related disturbance includes block failure, thaw slumping, and mechanical abrasion that thaws and mobilizes OC to the marine environment. Inundation-related disturbance includes thawing, seawater intrusion, and mobilization by wave action. We assume that OC is disturbed to a depth of 2 m below sea level in areas that are eroded or inundated and that disturbed OC stocks are no more than estimated OC stocks in the top 3 m.

We estimate that under medium and high emissions scenarios, erosion and inundation will by 2100 disturb 453 (367 to 524, 68% credible interval) and 562 (476 to 616) Tg OC, respectively, which is eight and eleven times the cumulative OC that erosion alone could disturb by 2100 (Fig. 3). Mean OC disturbance rates rise from 0.7 Tg C/y at 2020 to 11(14) Tg C/y at 2100 under medium(high) emissions. Our 2020 rates exceed, but are of similar magnitude to, previous estimates of present-day OC fluxes from Alaskan Beaufort Sea coastal erosion—0.16 Tg OC/y (31)—and by 2100 will be ∼40 times the present-day OC fluxes from the three largest rivers draining the ACP (∼0.3 Tg OC/y) (35). While quantitative conversion of our disturbed OC into greenhouse gas emissions exceeds this paper’s scope, if ∼1 to 10% becomes converted to CO_2_, atmospheric CO_2_ would rise ∼0.025 to 0.25 ppm by 2100 (36). Terrestrial OC degradation could further effect the regional marine ecosystem by tipping marginal Arctic seas from sinks to sources of atmospheric CO_2_s (e.g., 37), acidifying the ocean (38), altering marine productivity (39), and reshaping Beaufort Sea food webs (40). These impacts highlight the need to consider permafrost subsidence, sea-level rise, and erosion in projections of OC mobilization and transformation.

## 2. Future Arctic Coasts

Human activity is changing the Earth System fast enough that the recent past has lost predictive power as a template for the future (41). Instead, climate science disciplines are reaching deeper into the past to find analogues for the states of future Earth, the rates of future change, and the relative importance of the processes making that change.

We argue that portions of the Arctic shoreline will undergo transformative changes not only in state and rate—more land lost, increased erosion—but also in which processes drive change. For at least the last century, erosion has governed coastal change everywhere in the Arctic, save locations where glacial isostatic uplift dominates (42, Fig. 1*A*). We project that for the Beaufort Sea coast, this status quo will tip by midcentury as land loss due to the combination of inundation and erosion overtakes land loss due to erosion alone. This transition will likely also occur elsewhere in the Arctic. The shift will happen faster in areas far from ice sheets like the East Siberian, Laptev, and Barents Seas. However, areas with isostatic uplift will not be immune: Some parts of Northwest Svalbard have undergone net subsidence for the last century because of permafrost thaw (43). While erosion will continue to dominate in areas with high bluffs, such as the Alaskan Chukchi margin between Wainwright and Utqiaġvik, more and more of the Arctic will enter an inundation paradigm.

The consequences of this paradigm shift are hard to predict but will likely be profound. Little is known about how permafrost evolves when it is inundated versus eroded (44). Rapid inundation may insulate permafrost from increasingly high Arctic summer temperatures that, by season’s end, are degrading Pleistocene permafrost—a process that causes landscape-scale subsidence (19). This insulating effect will lessen, however, as mean annual Arctic Ocean bottom temperatures exceed 0 ^°^C—which they are projected to do throughout the Arctic by midcentury—and subsea permafrost thaws rapidly from above (45). Inundation could also change the fate of OC by shifting redox conditions: Eroded material is likely to degrade faster under aerobic conditions in the water column, whereas inundated material could degrade more slowly under anaerobic conditions in the subsurface. Alternatively, inundation may degrade more permafrost by covering it with salty brine that, as it percolates downward, will drive thaw by reducing the permafrost melting point (44)—degradation that may be intensified by storm surges if Arctic cyclone intensity and duration continue to increase, as they have since ∼1,950 (46). The future evolution of barrier islands is also uncertain: Rising seas may submerge them, exposing shorelines to erosive forces; or as barrier islands migrate inland they may intersect the coastline, causing unknown changes to permafrost subsidence.

Either way, an Arctic shoreline governed by inundation will pose new challenges to communities whose homelands—including infrastructure, hunting grounds, subsistence access routes, cultural heritage sites, landscapes, and the soil itself—are disappearing. Future research on Arctic shoreline evolution should be motivated by the needs of these communities, who will need support to respond to the paradigm shift in 21st-century Arctic coastal change that we project here.

## 3. Materials and Methods

Future ACP evolution is projected using a 5 m Alaska Digital Elevation Model (hereafter, DEM) based on InSAR source data of 5 m or higher resolution collected between 2012 and 2018 (47). We take 2015 as our simulation’s first year. The DEM is split into 78 overlapping subregions S∈DEM that encompass all coastal areas that in our maximum projections are inundated or eroded by 2100 (Fig. 2). Computations described below are performed on each subregion in isolation. Overlapping sections are then compared, and any pixel covered by ocean in either section is considered to be land replaced by ocean—a procedure that prevents double-counting.

Topography is defined as positive relief (H) in DEM areas above mean sea level in 2015:

[1] H(x,y)=DEM(x,y)·C(x,y),

where the ocean function C(x,y) is defined by

[2] $$\begin{array}{c} C\left(x,y\right)=\left\{\begin{array}{cc} 1 & \;\text{if DEM}(x,y)>0\\ NaN & \;\text{if DEM}(x,y)\leq 0 \end{array}\right., \end{array}$$

where NaN, short for “not a number,” denotes grid cells omitted from calculations. We note that (Eq. **1**) led to all terrestrial and lacustrine areas being correctly identified as land.

### 3.1. Lake Depth Correction.

The DEM represents freshwater lakes as flat areas whose elevation equals the unfrozen surface water elevation. To approximate lake bathymetry in these flat areas, we follow (27) (https://catalog.northslopescience.org/hr/dataset/2285), who found that North Slope lakes that froze completely in winter were 94% likely to be shallower than 1.6 m, while lakes that remained at least partly unfrozen were 98% likely to be more than 1.6 m deep. We derive an initial topography (T) by correcting elevation (H) for the depth of these lakes (L):

[3] $$T_{0}\left(x,y\right)=H_{0}\left(x,y\right)-L\left(x,y\right),$$

where lake depth L(x,y) is defined by

[4] $$\begin{array}{c} L\left(x,y\right)=\left\{\begin{array}{cc} 2.0\;meters & \;\text{if not frozen solid in winter}\\ 1.0\;meter & \;\text{if frozen solid in winter} \end{array}\right. \end{array}$$

Lake depths were derived from the median empirical frozen and unfrozen lake depth distributions from ref. 27. Because median lake depth exceeds 2 m, this correction likely leads us to underestimate lake depth overall and is therefore a conservative choice.

### 3.2. Sea-Level Change.

Relative sea level (RSL) change is estimated following projections from the 5th National Climate Assessment (NCA5 30). These projections account for RSL change due to several processes, including thermal expansion, the melting of mountain glaciers and the Greenland and Antarctic ice sheets, and vertical land motion (VLM), which encompasses regional processes like glacial isostatic adjustment [GIA, the gravitational, deformational, and rotational response of the solid Earth to changes in ice and liquid water loading (48)] and local processes like groundwater pumping. The NCA5 assesses VLM via a statistical model that converts tide-gauge observations into a spatially varying but temporally linear RSL change rate (16, 17). This assessment’s accuracy depends on tide gauge density. Long-term, high-quality tide gauge records are scarce in northern Alaska: The Permanent Service on Mean Sea Level includes only a single ACP gauge (Prudhoe Bay). The NCA5 projections’ 1-degree gridding also implies that processes driving nearshore VLM resemble those driving VLM on land. This assumption breaks down when interannual VLM is dominated by permafrost subsidence. Additionally, the Prudhoe Bay gauge cannot capture the spatial variability in ACP permafrost subsidence. For these reasons, it is unlikely the NCA5 RSL projections accurately represent present-day ACP VLM rates from permafrost subsidence. We therefore model that VLM component separately.

### 3.3. Permafrost Subsidence.

We estimate permafrost subsidence using an empirical approach. We aggregate interannual permafrost subsidence estimates from low-lying regions in Alaska, Arctic Canada, and Russia (*SI Appendix*, Fig. S1). To be included, a record must meet several criteria. First, it must span 3+ years. Second, records must be based off high-precision measurement, for instance, differential GNSS measurements repeated at the same time each year (20), differential GNSS combined with InSAR, thaw tube measurements, or repeat terrestrial laser scanning (rLiDAR) benchmarked by GNSS (12, 13, 18). Subsidence from GNSS—though not from InSAR, thaw tube, or other relative measurements—contains glacial isostatic adjustment, which the NCA5 sea-level estimates also include. However, this duplication is not an issue, as ACP GIA [0.1 to 0.3 mm/y, (49)] is much smaller than permafrost subsidence uncertainties (*SI Appendix*, Fig. S1). Third, records must describe the landscape type whose subsidence is measured. We map subsidence estimates onto Landsat-derived ACP landscape classifications (29). Landscape type correlates strongly with ground ice content (26) and late-season thawing of subactive layer ground ice (19). Since late-season ground ice thaw likely drives interannual landscape-scale permafrost subsidence (20)—and no subkilometer-scale ACP ground ice estimates exist—we use landscape type as a proxy for permafrost subsidence. We assume that present-day subsidence rates continue until 2100—a conservative choice given, for instance, warming ACP air temperatures and the probable expansion of ACP taliks in the 21st century.

### 3.4. Erosion.

Erosion (E) is estimated for each subregion following spatially varying projections from a semi-empirical model that combines climate reanalyses, observations, Earth system modeling, and ocean surface wave simulations (28). Erosion is initialized as 0 at 2015. For each subsequent year, the mean erosion projected by (28) for each subregion is added to the previous year’s erosional tally:

[5] $$E_{t}=E_{t-1}+\frac{\sum_{x=1}^{n}\sum_{y=1}^{m}E_{t}\left(x,y\right)}{n\cdot m},$$

where n and m are subregion dimensions. When $E_{t}$ exceeds 5, a threshold set by the 5 m DEM resolution, erosion initiates. Erosion is simulated by convolving a 3x3 cross-shaped kernel ($K_{e}$) across the subregion. Eroding regions—nonocean areas with sum >50% of the kernel sum, a threshold that isolates shorelines regardless of orientation—are reclassified as ocean:

[6] $$\begin{array}{c} S_{e}\left(x,y\right)=\left\{\begin{array}{cc} S(x,y) & \;\text{where}\;S\left(x,y\right)*K_{e}<\frac{1}{2}\sum K_{e}\\ NaN & \;\text{where}\;S\left(x,y\right)*K_{e}>\frac{1}{2}\sum K_{e}, \end{array}\right. \end{array}$$

where $S_{e}$ is a posterosion subregion. Erosion here resembles the erosional operator in mathematical morphology, a standard image processing tool.

By implementing (28), our erosion algorithm accounts for the main thermo-mechanical drivers of 21st-century erosion, namely temperature, sea ice, and ocean surface waves. However, it does not explicitly resolve coastal erosion itself. Rather, it relies on empirical relationships between erosion and its thermo-mechanical drivers. Physics-based, explicit models of coastal permafrost erosion first modeled niche evolution as an analytical function of ocean temperature, nearshore water depth, and inundation duration, then successively reproduced niche growth, bluff failure, slumping, wave propagation, thermodenudation, thermal abrasion, sediment transport, and other processes to project lateral cliff migration and vertical erosion of abutting beaches (50). These models are routinely applied to 2D shoreline transects but never expanded to 3D to project erosion at regional or climatic scales (51) due to impractical computational costs. We therefore employ this simpler algorithm as an approximation, which allows us to assess the relative importance of erosion, permafrost subsidence, and sea-level rise at regional and climatic scales. Our model also does not differentiate between erosion of barrier islands and mainland coast—a simplification that likely leads us to underestimate future erosion, as Arctic barrier islands shield coastlines from abrasive wave action and storm surge (52).

### 3.5. Storm Smoothing.

Storms periodically reshape ACP shorelines. We approximate this process via a procedure similar to Eq. **6**. We convolve a 10 × 10 boxcar kernel ($K_{s}$) across each subregion. Terrestrial coastal areas whose convolved sum is <50% the sum of $K_{s}$ are reclassified as ocean. Coastal ocean areas whose convolved sum exceeds half the sum of $K_{s}$ are reclassified as land with 1 m topographic relief:

[7] $$\begin{array}{c} S_{s}\left(x,y\right)=\left\{\begin{array}{cc} \emptyset & \;\text{where}\;S_{e}\left(x,y\right)*K_{s}<\frac{1}{2}\sum K_{s}\\ 1 & \;\text{where}\;S_{e}\left(x,y\right)*K_{s}>\frac{1}{2}\sum K_{s} \end{array}\right. \end{array}$$

This operation redistributes sediment along the coast with a smoothing lengthscale of 50 m. Modest changes in $K_{s}$ size were found to have negligible impact on our results. Beyond increasing erosion, future storms in the ACP will have other effects, including changing precipitation, which may acceleration surface degradation and landscape denudation. While estimating such processes exceeds this study’s scope, accounting for these effects should be a priority for future research.

### 3.6. Inundation.

Inundation converts coastal ACP regions at sea level into marine inlets. We model this by convolving a 10 × 10 circular kernel across each subregion to identify areas within 50 m of the coast. Areas <0.2 m above sea level in this zone—a threshold set by ACP tidal amplitudes—are reclassified as ocean. This protocol elides short-term nearshore processes that could dampen local postinundation erosion rates. However, on decadal timescales, erosional breaching of freshwater lakes, inundation, and subsequent erosion of former lake shorelines have been observed across the ACP (32). We therefore argue that immediate inundation is a reasonable approximation.

### 3.7. Infrastructure.

The fraction of infrastructure damaged by erosion and inundation is estimated using the infrastructure maps of the North Slope Science Initiative. We differentiate these maps into “Developed Areas” and “Roads” for cities, towns, and legacy sites—i.e., Distant Early Warning Line sites— and oilfields as well as oil pipelines. We consider developed area polygons damaged if they intersect with the ocean. Road polygons are damaged only at the specific locations where seawater covers them. These maps omit many sites that communities consider important; future work should assess impacts to other important sites, many of which are cataloged in the Traditional Land Use Inventory managed by the North Slope Borough (53).

### 3.8. Organic Carbon.

We quantify the OC disturbed by erosion and inundation by employing a 300 m circumpolar soil carbon dataset (54). Topography in subregion S at each timestep is compared to 2015 topography. OC is deemed disturbed at time t if the area is ocean at time t but had topography in 2015. OC disturbance is quantified by (54) in only the top 3 m of sediment, and we assume that all deeper sediment contains no OC. This choice likely leads us to underestimate OC disturbance, particularly in areas with high coastal relief.

Inundation is modeled as disturbing OC down to 2 m below sea level. Three factors determined this depth: tidal range, estimated as 10 to 20 cm; active layer thickness of inundated sediments, estimated as 30 to 40 cm; and historical patterns of nearshore erosion and deposition. From comparisons between 1945 to 1953 and 2012 to 2015 hydrographic surveys, (55) describes 0.5 to 3+ m of erosion beyond barrier islands and 0 to 0.5 m of deposition within lagoon systems. In future, heightened 21st-century storminess may increase lagoonal sediment disruption (52). Assuming 21st-century sediment disruption depths fall in the mid-range of historical ranges, 2 m of disruptive penetration by erosion is a conservative choice, particularly given this study’s biogeochemical focus on OC disruption, which here encompasses sediment redistribution as well as erosion. Furthermore, even where erosion disturbs little, inundation causes rapid changes in the shallow subsurface. For instance, hypersaline brines produced during sea-ice formation percolate through newly inundated permafrost, lowering sediment freezing temperatures and accelerating thaw even with <2 m of inundation (56). Sediment resuspension, temperature, redox conditions, organic matter quality, and other factors impact the rate in which disturbed OC is remineralized. Given these uncertainties, we use a few simple assumptions: 2 m bsl of OC is disturbed, and 1 to 10% of disturbed OC is remineralized to CO_2_-C (36).

## Supplementary Material

Appendix 01 (PDF)

Dataset S01 (CSV)

## Data Availability

Model outputs data have been deposited in Zenodo (10.5281/zenodo.11177226) (57).
