# Supplementary material for: Permafrost thaw subsidence, sea-level rise, and erosion are transforming Alaska’s Arctic coastal zone
Source: Proc Natl Acad Sci U S A. 2024 Dec 3;121(50):e2409411121. doi: 10.1073/pnas.2409411121 (PMC11648609; doi:10.1073/pnas.2409411121)
Supplement: Supplementary file 1 — Appendix 01 (PDF) [file pnas.2409411121.sapp.pdf]

PNAS

Supporting Information for

Permafrost thaw subsidence, sea-level rise, and erosion are transforming Alaska's Arctic coastal zone

Roger Creel, Julia Guimond, Benjamin Jones, David M. Nielsen, Emily Bristol, Craig Tweedie, Pier Paul Overduin

Roger Creel
E-mail: roger.creel@whoi.edu

This PDF file includes:

Fig. S1
Legend for Dataset S1
SI References

Other supporting materials for this manuscript include the following:

Dataset S1

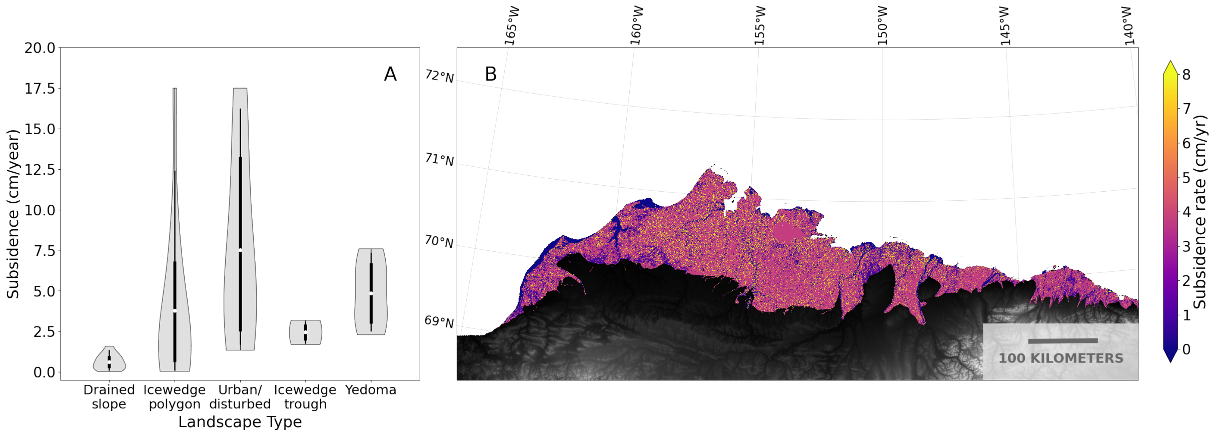

Fig. S1. Permafrost subsidence on Alaska's Arctic Coastal Plain (ACP) (A) Empirical estimates of permafrost subsidence from coastal Arctic landscapes. White dots denote means, vertical lines mark 66% and 95% confidence intervals. **(B)** Modeled permafrost subsidence based on mapping of empirical estimates to landscape classifications from (1).

SI Dataset S1 (627979_1_supp_10968332_sgptr5.csv)

Compilation of empirical rates of permafrost thaw subsidence from North America and Eurasia. Columns include ID, landscape type, landscape subtype, subsidence rate (m/yr), subsidence standard deviation, location, observation start (years CE), observation end (years CE), Elevation (m), and reference.

References

1. M Lara, I Nitze, G Grosse, A McGuire, Tundra landform and vegetation productivity trend maps for the Arctic Coastal Plain of northern Alaska. *Sci. Data* **5**, 180058 (2018).
